# VTQA: behavioral data from studies on compression ratios in vibrotactile signal processing

**DOI:** 10.1186/s13104-026-07874-8

**Published:** 2026-05-13

**Authors:** Evelyn Muschter, Lars Nockenberg, Shu-Chen Li, Eckehard Steinbach

**Affiliations:** 1 Centre for Tactile Internet with Human-in-the-Loop (CeTI), Technische Universität Dresden, Dresden, 01062 Germany; 2 Junior Research Group Human Perception and Action in Technology, Technische Universität Dresden, Dresden, 01062 Germany; 3 Chair of Lifespan Developmental Neuroscience, Faculty of Psychology, Technische Universität Dresden, Dresden, 01062 Germany; 4 Media Technology/Munich Institute of Robotics and Machine, Technische Universität München, München, 80333 Germany

**Keywords:** Vibrotactile signals, Compression ratios, Tactile codecs, Perceptual ratings, Human sensory experience

## Abstract

**Objectives:**

This dataset collection aims to facilitate research on the transmission of digitized vibrotactile signals over communication networks, emphasizing the importance of minimizing delay and bandwidth requirements while preserving perceptual quality in human sensory experiences.

**Data description:**

The dataset includes perceptual ratings derived from two user studies evaluating the quality of vibrotactile signals compressed using various single-point tactile codecs. Participants provided subjective quality ratings for a range of compression ratios to assess data fidelity and the extent to which compression algorithms may compromise quality in favor of file size reduction.

## Objective

The primary objective of this dataset collection is to provide insights into the perceptual impact of different compression methods on the quality of vibrotactile signals. The data were collected to evaluate how compression affects the fidelity of tactile experiences, which is crucial for the development of effective vibrotactile communication systems. Related publications include studies on perceptual quality assessment of compressed vibrotactile signals [1, 2].

## Data description

This dataset collection includes subjective similarity and quality ratings from 64 participants across two studies [1, 2]. The studies evaluated the perceptual effects of various vibrotactile signal compression methods and ratios. Key elements of the methodology include controlled experimental conditions, counterbalancing stimulus order, and precise timing of stimulus delivery. The dataset contains behavioral data, experimental conditions, and participant information, as well as stimuli and experimental code, providing a comprehensive resource for studying the impact of compression methods on perceived quality.

**Table 1:** Overview of data files and data sets

Label	File/data set name	Type	Description	Data repository (DOI)	Citation
Data file 1	README.md	File	Documentation file providing an overview and instructions for the dataset	Figshare ( (10.6084/m9.figshare.29504987))	[3]
Data file 2	CITATION.rtf	File	Citation information for the dataset collection	Figshare (10.6084/m9.figshare.29504987)	[3]
Data file 3	data_description.json	File	JSON file containing metadata that provides general information about the dataset collection	Figshare (10.6084/m9.figshare.29504987)	[3]
Data set 1	beh/	Folder	Collection of files related to behavioral data, including JSON and TSV files	Figshare (10.6084/m9.figshare.29504987)	[3]
Data file 4	beh.json	File	JSON sidecar file containing metadata for behavioral data stored in the beh/ folder	Figshare (10.6084/m9.figshare.29504987)	[3]
Data file 5	master_participants.json	File	JSON sidecar file with detailed descriptions of master_participants.tsv	Figshare (10.6084/m9.figshare.29504987)	[3]
Data file 6	master_participants.tsv	File	Tab-separated values file with participant-level metadata	Figshare (10.6084/m9.figshare.29504987)	[3]
Data set 2	study_1/	Folder	Folder containing all data files associated with study 1 [1]	Figshare (10.6084/m9.figshare.29504987)	[3]
Data set 3	study_2/	Folder	Folder containing all data files associated with study 2 [2]	Figshare (10.6084/m9.figshare.29504987)	[3]
Data set 4	stimuli/	Folder	Collection of files related to experimental stimuli used in the studies	Figshare (10.6084/m9.figshare.29504987)	[3]

*Table Description*: This table provides an overview of the data files and datasets included in the repository. The dataset collection is based on the Brain Imaging Data Structure (BIDS) standard [4] (Version 1.10.0) with respect to its format, content, and organizational principles. However, since the specifications do not explicitly address data from multiple studies, it does not fully adhere to all aspects of the BIDS specification, and as such, it includes a master_participants.tsv file in the root of the data, along with separate study-specific directories. Note: All referenced material in the table is available under the Creative Commons Attribution-NonCommercial-NoDerivatives 4.0 International License (CC BY-NC-ND 4.0)

### Key elements of the methodology

The experiments were conducted to evaluate subjective similarity quality ratings of vibrotactile stimuli under various compression conditions. Participants were presented with pairs of vibrotactile signals (test and reference) delivered to the pulp of their non-dominant index finger. The primary focus was to assess the perceptual impact of different compression ratios (CRs) and compression methods on the quality of the signals. Participants rated the similarity of the signals on a numeric scale, providing insights into the perceptual effects of compression methods. In particular three vibrotactile codecs [5–7] were investigated. The vibrotactile codecs compressed tactile reference data traces [8] (described in [9]) using distinct strategies. All three codecs are designed to exploit human tactile perception, ensuring that compressed signals remain subjectively indistinguishable from the original up to certain compression thresholds (i.e., CRs). Detailed methodological descriptions, including experimental setup and equipment, are available in the repository files for reproducibility.

## Limitations

- A defined subset of original signals and CRs were considered.
- Small sample sizes in some conditions may limit generalizability.
- Some CRs were missing for specific participants due to later additions to improve sampling rates.

## Data Availability

The data described in this Data note can be freely and openly accessed on Figshare under https://doi.org/10.6084/m9.figshare.29504987 [3]. Please see Table 1 and references for details and links to the data.
